# Palladium Nanoparticles Grafted onto Phytochemical Functionalized Biochar: A Sustainable Nanozyme for Colorimetric Sensing of Glucose and Glutathione

**DOI:** 10.3390/molecules28186676

**Published:** 2023-09-18

**Authors:** Aakhila Banu, Arnet Maria Antony, Balappa Somappa Sasidhar, Shivaputra A. Patil, Siddappa A. Patil

**Affiliations:** 1 Centre for Nano and Material Sciences, Jain (Deemed-to-be University), Jain Global Campus, Kanakapura, Bangalore 562112, Karnataka, India; aakhila.banu@jainuniversity.ac.in (A.B.); a.maria@jainuniversity.ac.in (A.M.A.); 2 Chemical Sciences & Technology Division, National Institute for Interdisciplinary Science & Technology (CSIR-NIIST), Thiruvananthapuram 695019, Kerala, India; drsasidharbs@niist.res.in; 3 Pharmaceutical Sciences Department, College of Pharmacy, Rosalind Franklin University of Medicine and Science, 3333 Green Bay Road, North Chicago, IL 60064, USA

**Keywords:** nanozyme, biochar, BC-AHE@Pd, H_2_O_2_ sensing, glucose, glutathione

## Abstract

The devising and development of numerous enzyme mimics, particularly nanoparticles and nanomaterials (nanozymes), have been sparked by the inherent limitations imposed by natural enzymes. Peroxidase is one of the enzymes that is extensively utilized in commercial, medical, and biological applications because of its outstanding substrate selectivity. Herein, we present palladium nanoparticles grafted on *Artocarpus heterophyllus* (jackfruit) seed-derived biochar (BC-AHE@Pd) as a novel nanozyme to imitate peroxidase activity en route to the rapid and colorimetric detection of H_2_O_2_, exploiting *o*-phenylenediamine as a peroxidase substrate. The biogenically generated BC-AHE@Pd nanocatalyst was synthesized utilizing *Artocarpus heterophyllus* seed extract as the reducing agent for nanoparticle formation, while the residue became the source for biochar. Various analytical techniques like FT-IR, GC-MS, FE-SEM, EDS, TEM, SAED pattern, *p*-XRD, and ICP-OES, were used to characterize the BC-AHE@Pd nanocatalyst. The intrinsic peroxidase-like activity of the BC-AHE@Pd nanocatalyst was extended as a prospective nanosensor for the estimation of the biomolecules glucose and glutathione. Moreover, the BC-AHE@Pd nanocatalyst showed recyclability up to three recycles without any significant loss in activity.

## 1. Introduction

Nanoparticles (NPs) are materials with unique features that set them apart from their bulk equivalents, with at least one dimension of 1–100 nm [1], and have been considered to be the fundamental units of nanotechnology [2,3,4]. Since the commencement of time, numerous industries and people have employed particles in this size range [5]. The NPs have unique chemical and physical traits as a result of their extensive surface area and nanoscale size [6]. Numerous biological applications, including enzymatic inhibition, transport, and sensing, can be influenced by the capability of NPs, as they can mimic receptors in their ability to interact with bio-macromolecules, enabling effective sensing [7,8]. Even though they have enormous features, they have drawbacks as well, like agglomeration. As a consequence of their high surface energy, NPs tend to be unstable and prone to aggregation, thereby reducing their activity. Because of this, stabilizing NPs is necessary. This can be achieved by exploiting inert supports as a possible way to minimize these above-mentioned boundaries, improve the overall surface area and dispersion of the NPs, and consequently enhance their activity [9,10]. These systems can be cataloged as the so-called supported metal NPs. The precise particle morphology (size and shape), metal dispersion, concentration, and electrical characteristics of the NPs in their host environment are directly influenced by the distinctive properties of supported metal nanoparticles [11,12,13]. One of the sources of stable and economic support is carbonaceous material, especially biochar (BC), a highly porous carbon-based material generated from the abundantly available biomass by pyrolysis [14]. The presence of a range of surface functional groups such as hydroxyl, carbonyl, amino groups, and others makes BC a suitable support system for functionalization and dispersion of metal NPs [15]. Additionally, the recovery and recycling of noble metals are essential factors in sustainable catalysis. The use of BC as support makes these processes simple, as the support can be burned off and the metal economically recovered [16].

Natural enzymes offer an extensive range of potential uses in the industrial, medical, and biological realms, among others, due to their elevated catalytic activity and substrate specificity. Despite their potential, they frequently experience inherent problems like high costs, poor stability, and challenges with recycling [17,18,19]. Researchers have spent a lot of time investigating synthetic enzyme mimics to address these downsides. Nanozymes are nanosystems with an enzyme-like catalytic property to mimic natural enzymes with better stability, durability, and tunability while being inexpensive [20]. Nevertheless, the significant difference in substrate specificity between nanozymes and enzymes leaves a wide berth for improvement [21]. Various NPs and nanocomposites have been explored for their peroxidase-like activity for the sensing of H_2_O_2_ using peroxidase substrates like 3,3′,5,5′-tetramethylbenzidine (TMB) and *o*-phenylenediamine (OPD) for the oxidation process [22]. The generation of colored products during the oxidation assists in the estimation of the H_2_O_2_ colorimetrically making the sensing process simplified. Additionally, the nanozyme property of the NPs can be extended to the detection and quantification of other organic compounds, biological molecules, and heavy metals [23].

Of the various biomolecules, glucose, a nutrient and metabolic intermediary, is of key interest [20,24]. Greater concentrations of glucose, particularly in diabetes mellitus, can cause serious complications, including heart and kidney disorders, blindness, etc., and thus the body must maintain glucose levels between 3 and 8 mM [25,26]. On the other hand, one of the most prevalent intracellular non-protein biothiols is glutathione, which is involved in a diversity of cellular processes such as xenobiotic metabolism, intracellular signal transduction, and gene regulation [27]. The abnormal levels of cellular glutathione can be directly correlated with liver damage, cancer, AIDS, leukocyte loss, and cardiac issues [28]. Therefore, it is crucial to have access to quick, dependable, and precise sensors for tracking the levels of these biomolecules in bodily fluids. Here, we report the synthesis of palladium (Pd) NPs dispersed on functionalized BC derived from *Artocarpus heterophyllus* (jackfruit) seeds (BC-AHE@Pd) as a nanocatalyst for the detection of the biomolecules glucose and glutathione. The nanozyme was synthesized in three facile steps. The extract of *Artocarpus heterophyllus* seeds was utilized as the reducing, stabilizing, and capping agents in the formation of the Pd NPs. The residue of the *Artocarpus heterophyllus* seeds obtained after extraction became the source for biomass-derived char, the BC [29]. The intrinsic peroxidase-like mimic of the BC-AHE@Pd nanocatalyst was employed for the sensing of H_2_O_2_ and further explored for its potential as a nanosensor for the quantification of glucose and glutathione.

## 2. Results and Discussion

### 2.1. Synthesis of BC-AHE@Pd Nanocatalyst

**Scheme 1:**
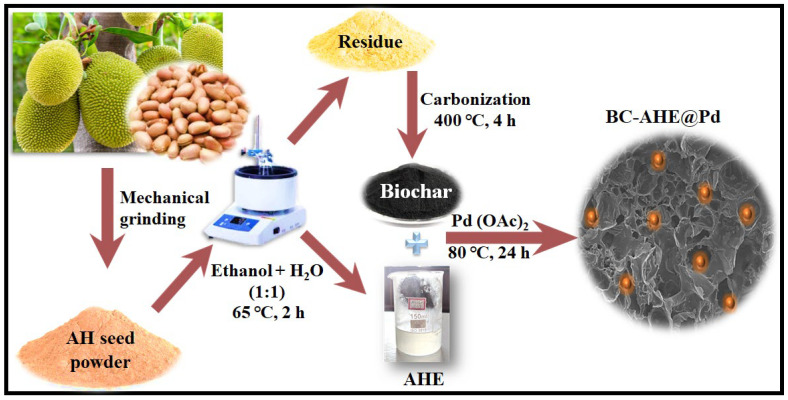
Synthesis of Pd NPs dispersed on functionalized BC derived from *Artocarpus heterophyllus* seeds (BC-AHE@Pd).

To vitalize the eco-friendly technique for producing Pd NPs, we reveal a biogenic approach for Pd NPs grafted on phytochemically functionalized BC derived from *Artocarpus heterophyllus* seeds as auxiliary material (BC-AHE@Pd). *Artocarpus heterophyllus* is an abundantly available non-exotic species that is widely cultivated for its fruit. The jackfruit seeds are mostly discarded, and utilizing them can reduce food waste. Additionally, their nutritional value can be used for diverse applications in industries such as cosmetics and medicine. The nanocatalyst was synthesized in a three-step process, as illustrated in Scheme 1. The steps involved are: (i) the extraction of *Artocarpus heterophyllus* seeds (AHE), (ii) pyrolysis of the dregs of the extract to obtain BC, and (iii) formation and dispersion of Pd NPs onto the BC to obtain the final BC-AHE@Pd nanocatalyst. The aqueous ethanolic AHE (1:1 *v*/*v*) contains phytochemicals that reduce Pd^2+^ to Pd^0^ and stabilize the formed NPs by forming a coating around them. Simultaneously, the BC was produced by the pyrolysis of the remains of the jackfruit seed after extraction at 400 °C for 4 h. The biomass tissue structure appeared to open up during the expansion phase of pyrolysis (~350 °C), with the stretching of the cell lumen and the cell wall until they were taut [30,31], undergoing diverse physical, chemical, and molecular changes [32]. Finally, the in-situ formation and grafting of the Pd NPs onto the BC was carried out in the presence of AHE to develop BC-AHE@Pd as a black-colored powder [33]. The availability of functional groups (C-O, C=O, COOH, OH, etc.) on the BC surface could be potentially used to bind the Pd NPs onto the BC support [34,35]. Furthermore, it is anticipated that the Pd NPs may experience phase segregation due to the catalyst’s support on highly porous carbon, such as BC [36]. This exceptional porosity in the support material fosters the formation of highly dispersed nanoparticles. Also, the functionalization of the BC with phytochemicals assists in the better adhesion of the Pd NPs through the stronger interaction of the functional groups present on the phytochemicals and the metal site. This may enhance the catalyst’s performance and stability by reducing the possible leaching of the Pd NPs.

### 2.2. Spectroscopic and Microscopic Analysis of BC-AHE@Pd

#### 2.2.1. GC-MS and Qualitative Analysis of AHE

The existence of phytochemicals in the AHE extract was confirmed with the help of GC-MS analysis as well as qualitatively. The GC-MS spectra of the ethyl acetate extract of AHE indicated the presence of 2-[3-(carbamoylmethyl)-2,4,6-trimethylphenyl]acetamide, 2,4-di-*tert*-butylphenol, 1-octadecanol, 1-hexacosene, ethylcyclooctadecane, and bis(2-ethylhexyl)phthalate (Figure S1). Similarly, through qualitative analysis, we observed the presence of glycosides, terpenoids, sugars, alkaloids, and saponins in the AHE extract (Table S1). The corresponding color changes associated with the tests are provided in Figure S2. The aforementioned phytochemicals are responsible for the successful reduction of Pd^2+^ to Pd^0^ to establish a stable BC-AHE@Pd as a nanocatalyst.

#### 2.2.2. UV–Visible Analysis

**Figure 1:**
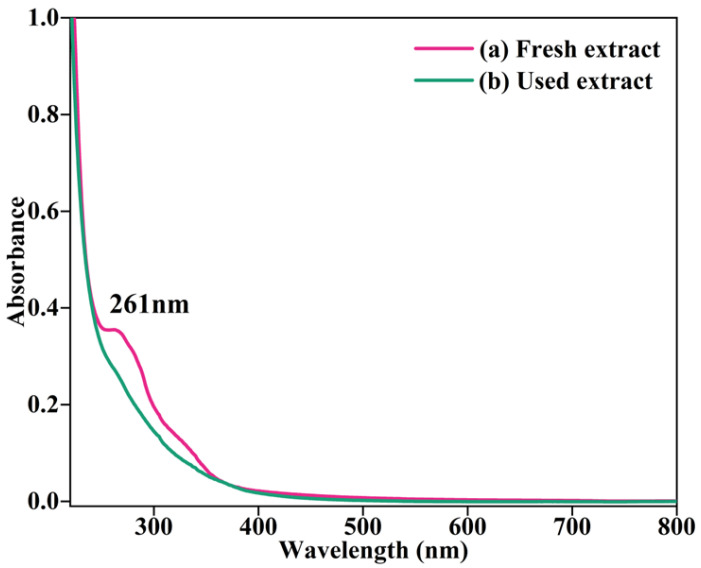
UV–visible spectra of AHE: (**a**) fresh and (**b**) after synthesis of BC-AHE@Pd.

At the start, the fresh AHE was exposed to UV–visible analysis to recognize its expediency in the development of BC-AHE@Pd nanocatalyst. The presence of terpenoids and other phytochemicals in the AHE extract was confirmed by the absorption peaks in the range of 240–320 nm (Figure 1a). This may be because of the n-π* and π-π* transitions taking place in the phytochemicals present in the AHE extract [37]. Analysis of the extract after the Pd^2+^ to Pd^0^ reduction reveals that the corresponding peaks that were present in the fresh extract have vanished (Figure 1b) demonstrating their utilization in the synthesis of the BC-AHE@Pd nanocatalyst.

#### 2.2.3. FT-IR Spectroscopy

**Figure 2:**
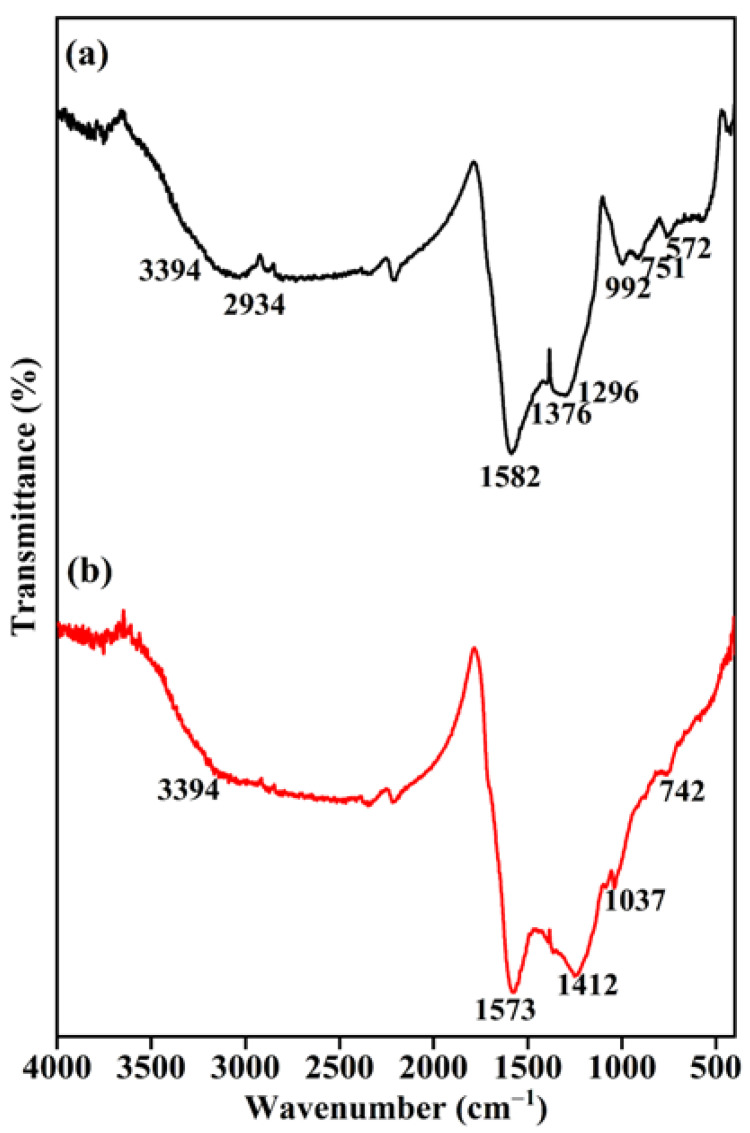
FT-IR spectra of: (**a**) BC and (**b**) BC-AHE@Pd.

To figure out the functional groups present in the prepared BC and final BC-AHE@Pd nanocatalyst, we executed FT-IR analysis. In the FT-IR spectrum of BC (Figure 2a), the broad-spectrum range of 3394–3521 cm^−1^ represents the O-H stretching (hydroxyl groups: alcoholic and phenolic) [38,39]. The peak at 2934 cm^−1^ can be perceived to be the distinctive C-H stretching vibrations of the aliphatic groups [40,41]. The absorbance peaks displayed at 1582, 1376, and 1296 cm^−1^ are due to the C=O, aromatic vibrations, and bending mode of vibration of the -NH_2_ molecule [38,42,43]. The band observed at 992 cm^−1^ is because of C-O stretching, and the peak detected at 751 and 572 cm^−1^ is due to the out-of-plane bending of the amine group [44]. The retention of the aforementioned different absorption peaks was seen in the synthesized BC-AHE@Pd nanocatalyst, while there was a slight shift in peak values. The functional groups, particularly the phenolic group, might interact with the surfaces of NPs through electron interactions [45]. Around 400 °C, aliphatic methyl, methylene, and methoxy groups can fracture, reforming into other functional groups like carbonyl and carboxyl, which can also interact with the NPs [43]. This suggests that there can be interactions between the functionalized BC support and the Pd NPs; thus, the formation of a novel BC-AHE@Pd nanozyme was established.

#### 2.2.4. *p*-XRD Analysis

**Figure 3:**
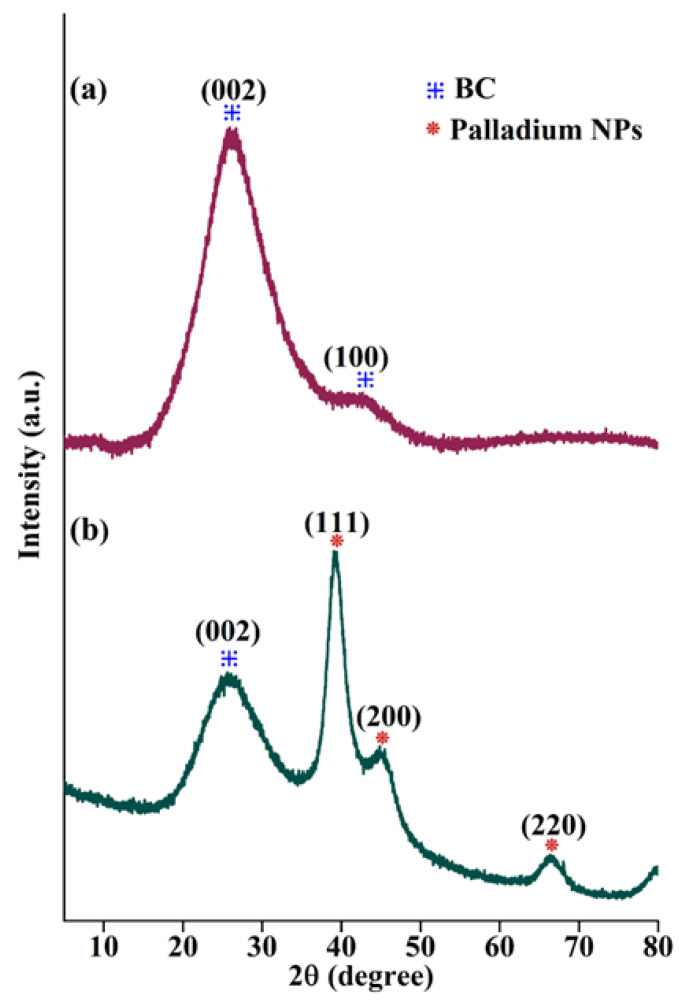
*p*-XRD pattern of (**a**) BC and (**b**) BC-AHE@Pd.

The *p*-XRD analysis was employed to depict the diffraction pattern of the BC support and the BC-AHE@Pd nanocatalyst. The diffraction pattern of the synthesized BC (Figure 3a) displayed broad peaks at 2θ values of 26.12° and 42.44°, corresponding to the reflection planes (002) and (100). The (002) plane relates to the aromatic carbonized structure, and the (100) plane can be assigned to the graphitic carbon [43]. In the diffraction pattern of the BC-AHE@Pd nanocatalyst (Figure 3b), the C(002) was retained while the C(100) disappeared due to the sharp diffraction peaks of Pd NPs. The diffraction peaks at 39.36°, 45.24°, and 66.68° are related to the (111), (200), and (220) reflection planes of Pd NPs in the face-centered cubic structure (JCPDS card no. 05-0681) [38]. The broadening of the Pd NP peaks may be attributed to the presence of BC in higher amounts when compared to the Pd quantity in the BC-AHE@Pd nanocatalyst. The average crystallite size of the Pd NPs was calculated from the Scherrer equation to be 2.89 nm. The smaller size of the Pd NPs may boost the activity of the system.

#### 2.2.5. FE-SEM Analysis

**Figure 4:**
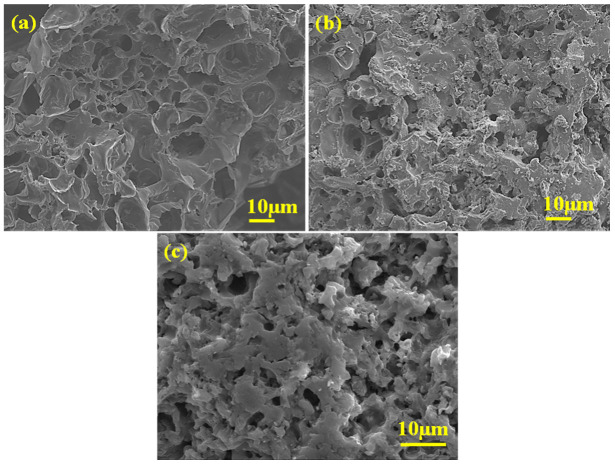
FE-SEM images of: (**a**) BC and (**b**,**c**) BC-AHE@Pd.

By making use of FE-SEM analysis, the surface morphologies of the synthesized BC support and BC-AHE@Pd nanocatalyst were explored. The investigation shows that the BC support had a porous morphology (Figure 4a), and the morphology was also well-retained in the generated BC-AHE@Pd nanocatalyst (Figure 4b,c). However, the nanocatalyst showed smaller pores, which may have occurred during the grafting of the Pd NPs. This apparent texture of the BC-AHE@Pd nanocatalyst can be linked to the coating of the phytochemicals and the produced Pd NPs altering the BC surface.

#### 2.2.6. EDS Analysis

**Figure 5:**
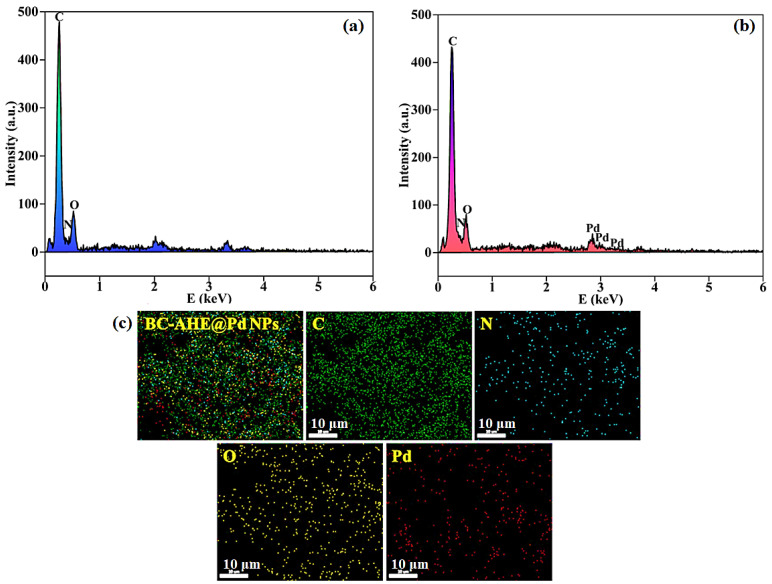
EDS spectra of: (**a**) BC, (**b**) BC-AHE@Pd NPs, and (**c**) elemental mapping of BC-AHE@Pd.

For the BC support and BC-AHE@Pd nanocatalyst, the elemental constitution and their dispersion were investigated by EDS analysis. According to the EDS spectrum, C, N, and O each have their own distinctive signals for BC and BC-AHE@Pd nanocatalyst (Figure 5a,b). Additionally, Pd is seen in the BC-AHE@Pd nanocatalyst (Figure 5b). The BC-AHE@Pd nanocatalyst’s elemental mapping (Figure 5c) also showed that these elements were distributed uniformly throughout the prepared nanocatalyst.

#### 2.2.7. HR-TEM Analysis

**Figure 6:**
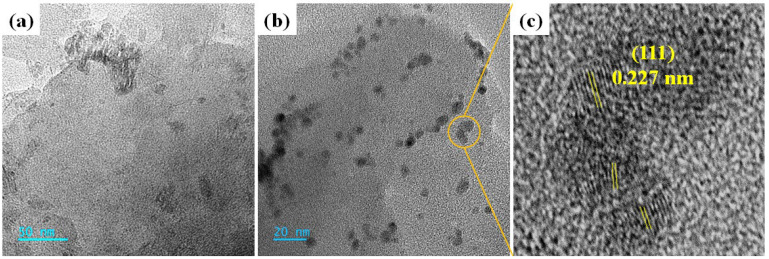
HR-TEM images of: (**a**) BC and (**b**,**c**) BC-AHE@Pd [inset c: d-spacing of Pd NPs].

The morphology of the synthesized BC support and BC-AHE@Pd nanocatalyst was further validated by HR-TEM analysis. The HR-TEM image of BC displayed the layered structure (Figure 6a), which was found to be retained even in the BC-AHE@Pd nanocatalyst with the more or less spherical-shaped Pd NPs dispersed on it (Figure 6b). A d-spacing of 0.227 nm was obtained, which was in agreement with the reflection plane (111) of the face-centered cubic structure of the Pd NPs (Figure 6c). Thus, we could conclude that the Pd NPs successfully adhered to the BC support.

#### 2.2.8. ICP-OES Analysis

ICP-OES analysis was performed to quantify the Pd content in the BC-AHE@Pd nanocatalyst. The analysis showed that the BC-AHE@Pd nanocatalyst had a Pd loading of 5.57% *w*/*w*.

### 2.3. Peroxidase-like Activity of BC-AHE@Pd in H_2_O_2_ Sensing

**Scheme 2:**
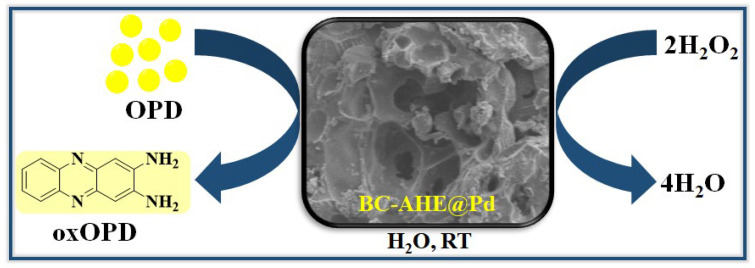
The peroxidase-like activity of BC-AHE@ Pd in sensing of H_2_O_2_ using OPD.

To scrutinize the capability of the synthesized BC-AHE@Pd nanocatalyst as an artificial enzyme (nanozyme), its peroxidase-like activity was carried out. The oxidation of peroxidase substrate OPD by H_2_O_2_ was performed at room temperature in acetate buffer (0.2 M, pH 4) (Scheme 2) [46], and the oxidation process was monitored using a UV-visible spectrophotometer.

**Scheme 3:**
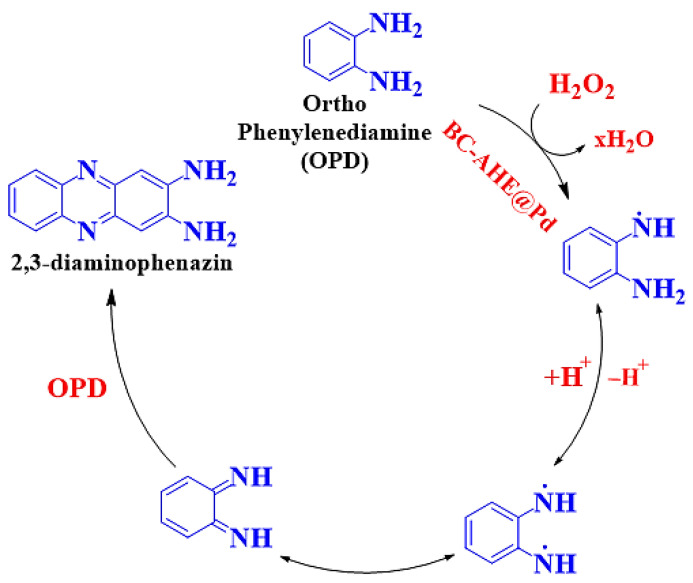
The radical mechanism of peroxidase-like activity of BC-AHE@ Pd.

**Figure 7:**
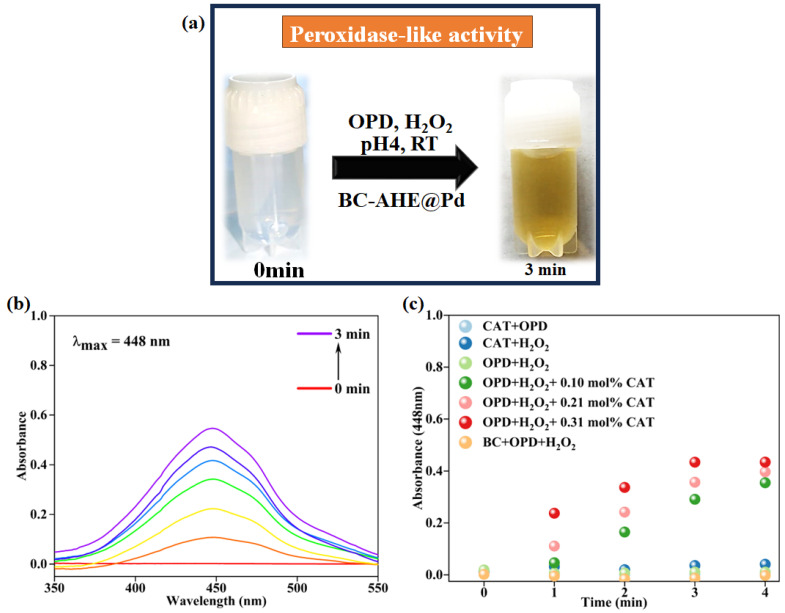
(**a**) Change in color of solution at time 0 and 3 min for the peroxidase-like activity of BC-AHE@Pd nanocatalyst, (**b**) time-dependent absorption spectrum of oxidation of OPD by H_2_O_2_ using BC-AHE@Pd NPs, and (**c**) plot of absorbance versus time under various reaction conditions of peroxidase-like activity of BC-AHE@Pd nanocatalyst (BC-AHE@Pd denoted as CAT).

Initially, both substrates (H_2_O_2_ and OPD) were colorless, and the oxidized product 2,3-diaminophenazine (oxOPD) was yellow-colored, with maximum absorbance found at 448 nm (Figure 7a,b). The inherent peroxidase-like activity of the BC-AHE@Pd nanocatalyst was investigated by performing control experiments. In the absence of either H_2_O_2_ or OPD, the oxidation process did not proceed. In the absence of the BC-AHE@Pd nanocatalyst, the progression was deliberately slow. This might have been affected by the small amount of ·OH radicals that were formed. With the addition of the BC-AHE@Pd nanocatalyst, we discerned the oxidation process taking place wildly in reduced time. In addition, we observed an increase in activity paralleled with a sharp increase in slope with an increase in the catalyst loading from 0 to 0.37 mol% Pd of the BC-AHE@Pd nanocatalyst (Figure 7c). In the presence of the BC-AHE@Pd nanocatalyst, H_2_O_2_ could produce hydroxyl radicals (**·**OH) easily, which converted to H_2_O by oxidizing the substrate OPD to oxOPD. The radical mechanism for the oxidation of OPD is represented in Scheme 3 [47].

#### 2.3.1. Effect of Reaction Parameters on the Peroxidase-like Activity of BC-AHE@Pd

**Figure 8:**
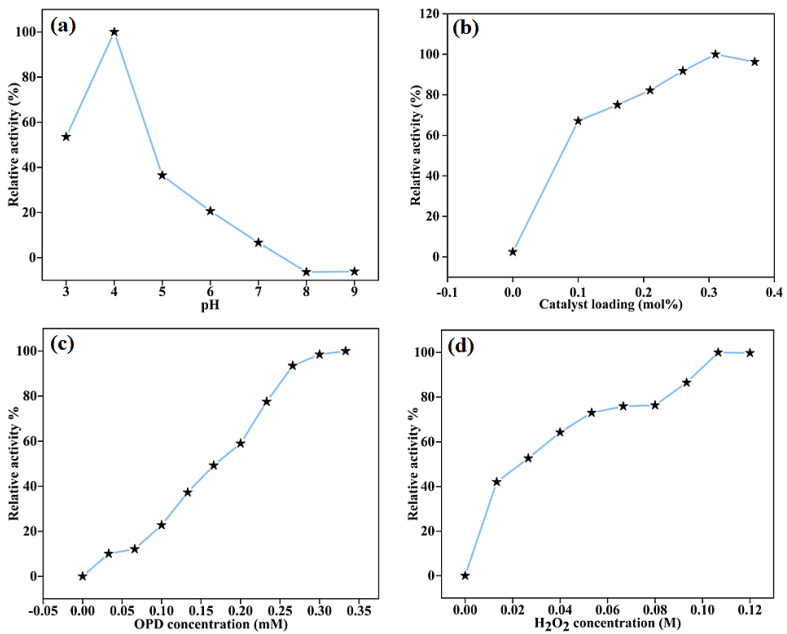
Percent relative activity for the peroxidase-like activity of BC-AHE@Pd with varying (**a**) pH, (**b**) catalyst loading, (**c**) OPD concentration, and (**d**) H_2_O_2_ concentration.

To determine the effect of reaction parameters such as variation in pH of acetate buffer, catalyst loading, and concentrations of H_2_O_2_ and OPD on the peroxidase-like activity of the BC-AHE@Pd nanocatalyst, the oxidation was carried out under varying reaction conditions. The peroxidase-like activity is related to the pH according to the pH-dependent studies, as well as the concentrations of substrates and nanocatalysts [48,49]. The superlative activity of the BC-AHE@Pd nanocatalyst was obtained at pH 4 (Figure 8a), with OPD (100 μM) and H_2_O_2_ (40 mM) in the presence of 0.31 mol% Pd of the BC-AHE@Pd nanocatalyst. Higher pH resulted in lesser oxidation, which may have been caused by a reduced amount of H_2_O_2_ breakdown [38]. The examination was further expanded to optimize the BC-AHE@Pd nanocatalyst loading for the best oxidation conditions while retaining the same concentrations of OPD and H_2_O_2_ at pH 4. The oxidation process was improved, as expected, with increasing nanocatalyst loading from 0 to 0.31 mol% Pd) (Figure 8b). Additionally, the relative activity percentages for the substrates OPD (0–100 μM) and H_2_O_2_ (0–40 mM) were carried out individually while maintaining the concentration of the counter substrate constant at pH 4 with a catalyst loading of 0.31 mol% Pd of BC-AHE@Pd nanocatalyst. The activity increased with an increase in the substrate concentrations (Figure 8c,d).

#### 2.3.2. Kinetic Analysis of the Peroxidase-like Activity of BC-AHE@Pd

**Figure 9:**
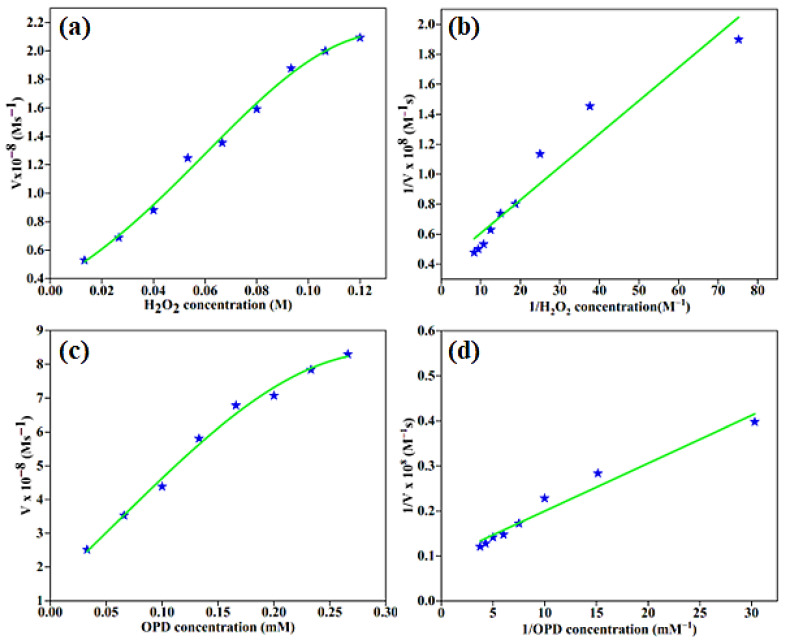
Steady-state kinetics and Lineweaver–Burke plot for the peroxidase-like activity of BC-AHE@Pd for: (**a**,**b**) H_2_O_2_ (with 83.33 μM OPD and 0.013–0.12 M H_2_O_2_) and (**c**,**d**) OPD (with 33.33 mM H_2_O_2_ and 0.033–0.266 mM OPD).

The kinetic parameters of the enzyme–substrate affinity for the BC-AHE@Pd nanocatalyst with the substrates OPD and H_2_O_2_ were calculated using the Michaelis–Menten equation and Lineweaver–Burk plot (Equations (1) and (2)).

(1) $$V_{0}=\frac{V_{max}\left[S\right]}{K_{m}+\left[S\right]}$$

(2) $$\frac{1}{V_{0}}=\frac{K_{m}}{V_{max}\left[S\right]}+\frac{1}{V_{max}}$$

where V_0_ and V_max_ are the initial and maximum rates of conversion with a substrate concentration [S], and K_m_ is the Michaelis constant. The absorbance values of OPD and H_2_O_2_ were fitted into the aforementioned equations to calculate the K_m_ and V_max_ values (Figure 9). They were found to be 57.32 and 0.114 mM for K_m_ and 2.59 × 10^−8^ and 10.72 × 10^−8^ Ms^−1^ for V_max_ obtained for H_2_O_2_ (R^2^ = 0.926) and OPD (R^2^ = 0.963), respectively. Likewise, the limit of detection (LOD) for H_2_O_2_ and OPD were found to be 0.1115 M and 0.0804 mM, respectively.

#### 2.3.3. Catalyst Reusability

**Figure 10:**
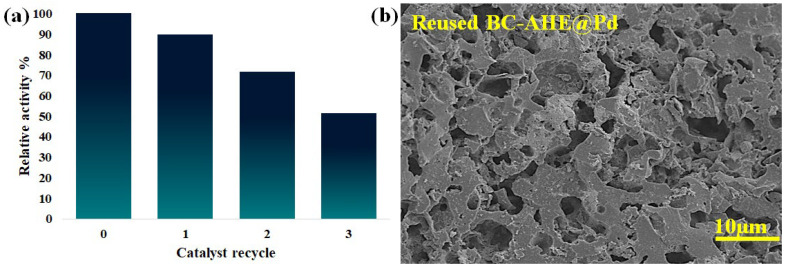
(**a**) Recyclability of BC-AHE@Pd in the oxidation of OPD with H_2_O_2_ and (**b**) FE-SEM image of the three-times recycled BC-AHE@Pd.

A catalyst’s marketable significance is improved by its capacity to be recycled and reused once again without losing its catalytic activity. The generated BC-AHE@Pd nanocatalyst was subjected to a recyclability study for its peroxidase-like activity in the oxidation of OPD. The heterogeneity of the BC-AHE@Pd nanocatalyst, being insoluble in the reaction medium, made the recovery of the nanocatalyst facile by simple centrifugation of the reaction mass. The recovered nanocatalyst was dried overnight at 80 °C and further used for the next cycles to investigate its reusability. The BC-AHE@Pd nanocatalyst was reused up to three recycles, and its relative activity is depicted in Figure 10a. From the relative activity (%) graph of recyclability, the recyclability of the BC-AHE@Pd nanocatalyst was found to decrease by ~50% in the third cycle, and it could be because of aggregation/agglomeration of the NPs. The three-times recycled BC-AHE@Pd nanocatalyst was subjected to FE-SEM analysis to inspect any change in its surface morphology. The FE-SEM image (Figure 10b) showed retention in the surface morphology to that of the fresh nanocatalyst (Figure 4b), exhibiting the nature of the nanocatalyst to be intact.

#### 2.3.4. Comparison of Performance of BC-AHE@Pd

To evaluate the competence of the synthesized BC-AHE@Pd nanocatalyst in peroxidase-like activity, the K_m_ and V_max_ values were compared with other heterogeneous catalysts reported in the literature (Table 1). The performance of the BC-AHE@Pd nanocatalyst was found to be comparable, with scope for further improvement.

### 2.4. Sensing of Glucose Using BC-AHE@Pd

**Scheme 4:**
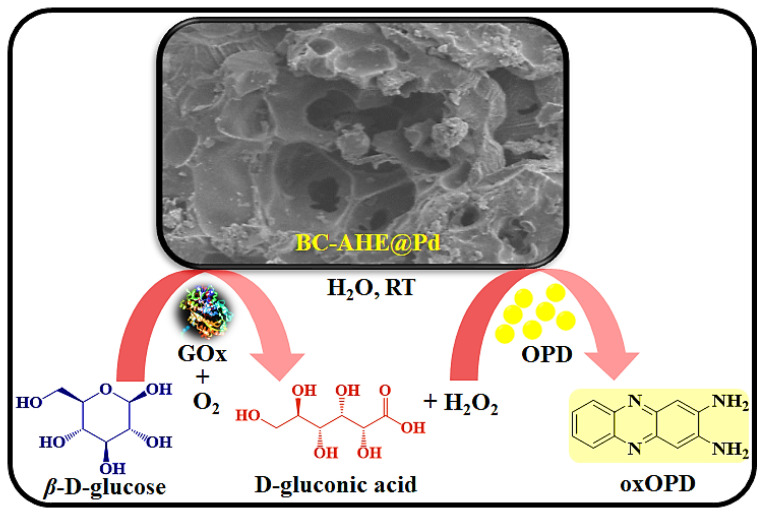
Schematic illustration of the colorimetric detection of glucose using BC-AHE@Pd.

**Figure 11:**
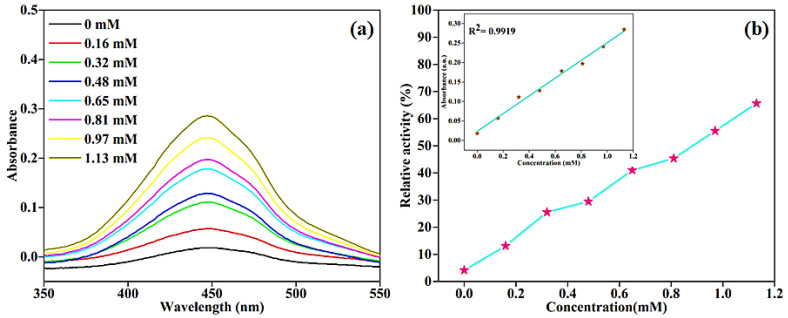
(**a**) The change in absorbance with different concentrations of glucose and (**b**) relationship between % relative activity and concentration of glucose (inset: linear relationship plot of glucose).

For the detection of glucose, colorimetric assays have been developed worldwide due to their remarkable advantages, such as low cost, high sensitivity, and ease of use [53,54]. Since the glucose oxidase enzyme (GOx) has a higher level of glucose selectivity, it is employed as a reference enzyme for biosensors [55]. To highlight the promising exertion of the BC-AHE@Pd nanocatalyst, we executed a colorimetric study for the sensing of glucose to detect and quantify glucose levels. The Pd NPs on the surface of the BC-AHE@Pd nanocatalyst bind with the GOx. The glucose molecules encountered on the surface of the BC-AHE@Pd nanocatalyst interact with the GOx, leading to the oxidation of glucose to gluconic acid in the presence of O_2_, accompanied by the evolution of H_2_O_2_. The generated H_2_O_2_ oxidizes the OPD substrate to oxOPD (Scheme 4). With the increase in glucose concentration (0–1.13 mM), a linear increase in absorbance at 448 nm was seen, and the observed color change was recorded using a UV–visible spectrophotometer (Figure 11a). The range of linearity for glucose was found to be 0.16–1.13 mM, with a LOD of 7.46 μM (Figure 11b). Further, the ability of the BC-AHE@Pd nanocatalyst in glucose sensing was compared for its sensitivity with other reported nanozymes (Table 2).

### 2.5. Sensing of Glutathione Using BC-AHE@Pd

**Scheme 5:**
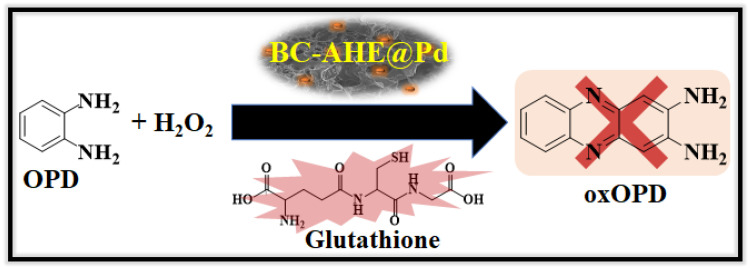
Schematic illustration for the colorimetric detection of glutathione using BC-AHE@Pd.

**Figure 12:**
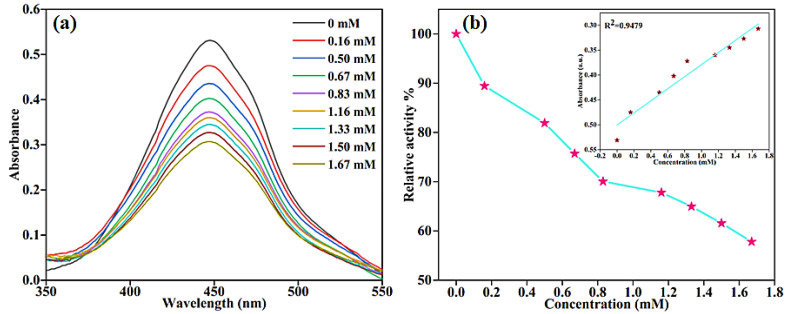
(**a**) The change in absorbance with different concentrations of glutathione and (**b**) relationship between % relative activity and concentration of glutathione (inset: linear relationship plot of glutathione).

Having successfully explored the peroxide-like activity of the BC-AHE@Pd nanocatalyst in glucose sensing, we broadened the scope of the nanocatalyst by investigating its potential in the detection of glutathione. The biothiol can be quantified by its inhibition property, whereby it binds to the surface of the BC-AHE@Pd nanocatalyst and is easily oxidized by H_2_O_2_ to the disulphide form [59]. Thus, the unavailability of H_2_O_2_ in oxOPD formation results in a substantial decrease in absorbance, leading to a possible quantification of glutathione (Scheme 5). As the glutathione concentration was raised (ranging from 0 to 1.67 mM), a proportional decrease in absorbance at 448 nm was observed and recorded utilizing a UV–visible spectrophotometer (Figure 12a). The linear range for glutathione’s response spanned from 1.16 to 1.67 mM, with the LOD determined to be 22.14 µM (Figure 12b). Alike glucose sensing, the BC-AHE@Pd nanocatalyst was compared for its competence in glutathione sensing with other reported literature (Table 3).

## 3. Experimental

### 3.1. Materials

The *Artocarpus heterophyllus* (jackfruit) seed was obtained from Tumkur district, Karnataka, India. The seeds were thoroughly washed with tap and distilled water consecutively, and then fragmented. The dried fragments were ground by mechanical grinding and then by mortar and pestle. Pd(OAc)_2_ (99% pure), CH_3_COOH, CH_3_COONa, OPD (extra-pure AR, 99%), 30% H_2_O_2_, D-(+)-glucose (≥99.5%), L-glutathione, GOx (ex. *Aspergillus niger*-10 K Units), and solvents (LR grade) were purchased from Avra, SRL, and Sigma-Aldrich chemical companies and were used as such. Heating was executed using a silicone oil bath and stirring with a magnetic stirrer. Unless explicitly stated, all reactions were carried out in an aerobic environment at room temperature.

### 3.2. Instrumentation and Analyses

Gas chromatography–mass spectrometry (GC–MS) analysis was performed by Shimadzu QP 2010SE GCMS (Kyoto, Japan, Ver. 2.6). Fourier transform infrared (FT-IR) spectroscopy was observed with a PerkinElmer Spectrum Two spectrometer (Hong Kong, China). To obtain the diffraction pattern and crystal structure, powder X-ray diffraction (*p*-XRD) was performed using a Rigaku X-ray diffraction Ultima-IV (Tokyo, Japan). Field-emission scanning electron microscopy (FE-SEM) and energy-dispersive X-ray spectroscopy (EDS) were used to investigate the morphology and elemental compositions individually and examined using a JEOL model JSM7100F (Tokyo, Japan). High resolution transmission electron microscopy (HR-TEM) images were obtained using a Jeol/JEM 2100 microscope (Tokyo, Japan). The amount of Pd in the nanocatalyst was determined with a Perkin Elmer Optima 5300 DV (Hong Kong, China) inductively coupled plasma optical emission spectroscopy (ICP-OES). The UV–visible spectra of extracts and the peroxidase-like activity for H_2_O_2_ sensing and colorimetric sensing of glucose and glutathione were carried out using a UV–visible spectrophotometer (UV-1900, Shimadzu, Kyoto, Japan) from the 200 to 800 nm wavelength range.

### 3.3. Extraction of Artocarpus heterophyllus Seeds (AHE)

The *Artocarpus heterophyllus* seed powder (2 g) was taken and dispersed in distilled water and ethanol in a ratio of 1:1 (*v*/*v*, 200 mL). Further, it was stirred for 2 h at 65 °C. After cooling to room temperature, the AHE was collected after centrifugation at a RCF of 1086.7× *g* and filtration. The AHE was stored in the refrigerator at 4 °C for further usage. The residual seed powder was dried at 80–85 °C for 12 h.

### 3.4. Preparation of Biochar (BC)

The dried residual seed powder after extraction (3 g) was pyrolyzed in a silica crucible at 400 °C for 4 h at a heating rate of 5 °C min^−1^. After cooling to room temperature, the carbonized biomass (BC) was obtained as a black-colored powder.

### 3.5. Synthesis of Pd NPs Grafted onto Phytochemical Functionalized BC (BC-AHE@Pd)

To the BC (1.5 g), AHE (150 mL) and Pd(OAc)_2_ (1.88 mmol) were added to obtain a 10 wt.% Pd loading onto the BC support and stirred at 80 °C for 24 h. Upon attaining room temperature, the formed BC-AHE@Pd nanocatalyst was isolated and washed with distilled water (2 × 20 mL) and ethanol (1 × 20 mL). The black-colored residue was dried overnight at 80 °C. It was then ground and stored in a container for further use.

### 3.6. Peroxidase-like Activity of BC-AHE@Pd

For the peroxidase-like activity of the BC-AHE@Pd nanocatalyst, each of the solutions was prepared in distilled water. The reaction was executed in a quartz cuvette. The reagents OPD (0–1.0 mM, 250 µL), H_2_O_2_ (0–0.4 M, 250 µL), and BC-AHE@Pd (0.31 mol% Pd) were added to acetate buffer (pH 4, 1.5 mL). The volume of the solution was made up to 2.5 mL using distilled water. The peroxidase-like activity was tracked by measuring the absorbance of the reaction system at selected time periods.

### 3.7. Recovery and Recycling of BC-AHE@Pd

Following the completion of the oxidation reaction, the BC-AHE@Pd nanocatalyst was separated from the reaction mixture by centrifugation at a RCF of 1086.7× *g*. The recovered nanocatalyst was rinsed with distilled water (2 × 15 mL) and ethanol (1 × 15 mL). After drying overnight at 80 °C, the nanocatalyst was used as such in subsequent recycles.

### 3.8. Glucose Sensing Using BC-AHE@Pd

Aqueous glucose solution (0–1.67 mM, 1 mL) and GOx (1 mg/mL, 100 μL) were incubated for 30 min. To this, acetate buffer (pH 4, 1.5 mL), 0.31 mol% Pd of BC-AHE@Pd nanocatalyst, and OPD (1 mM, 500 μL) were introduced. Following incubation for 10 min, the absorbance of the resultant system was measured.

### 3.9. Glutathione Sensing Using BC-AHE@Pd

For the detection of glutathione, to acetate buffer (pH 4, 1.5 mL), 0.31 mol% Pd of BC-AHE@Pd nanocatalyst, H_2_O_2_ (0.4 M, 500 μL), and glutathione (0–1.5 mM) were added, and the volume was made up to 2.5 mL with distilled water. The reaction mixture was incubated for 15 min, following which OPD (1 mM, 500 μL) was introduced. The mixture was further incubated for 5 min and its absorbance was measured.

## 4. Conclusions

Concisely, the sustainable BC-AHE@Pd nanocatalyst was prepared through an unpretentious methodology by the use of green solvents and procedures. The produced BC-AHE@Pd nanocatalyst was characterized thoroughly to confirm its chemical structure, surface morphology, elemental composition, and functional groups. The BC-AHE@Pd nanocatalyst was exploited for its peroxidase-like activity in H_2_O_2_ sensing using the substrate OPD, where it showed excellent activity. The BC-AHE@Pd nanocatalyst was easily recovered and thus recycled for up to three recycles. The decrease in activity may be attributed to the agglomeration of the nanocatalyst. Subsequently, it was subjected to the colorimetric detection and quantification of the biomolecules glucose and glutathione. The BC-AHE@Pd nanocatalyst showed good performance in the sensing of the biomolecules, with a promising future for nanosensor fabrication for real-time applications. Also, the BC-AHE@Pd nanocatalyst is highly stable, inexpensive, and eco-friendly. The BC-AHE@Pd nanocatalyst can be further explored for its application in organic transformations, electrocatalytic activity, wastewater treatment, etc.

## Figures and Tables

**Table 1:** Comparison of BC-AHE@Pd with other nanozymes with respect to OPD substrate.

Catalyst	K_m_ (mM)	V_max_ × 10^−8^ (Ms^−1^)	Reference
dendrimer-1-PdNPs	3.02	0.149	[50]
Fe_3_O_4_@Cu@Cu_2_O	0.85	13.1	[51]
Cu-MOF	0.54	7.87	[52]
**BC-AHE@Pd**	**0.113**	**10.72**	**Present work**

**Table 2:** Comparison of BC-AHE@Pd with other nanozymes for glucose detection.

Catalyst	Linear Range	LOD	Reference
Au-Ag-Pt	0–10 mM	289.6 µM	[56]
CoO-OMC	0–500 mM	68 µM	[57]
LA-MCM-41	50–1000 µM	37.5 µM	[58]
**BC-AHE@Pd**	**0.16–1.13 mM**	**7.46 µM**	**Present work**

**Table 3:** Comparison of BC-AHE@Pd with other nanozymes for glutathione detection.

Catalyst	Linear Range	LOD	Reference
Cy-AuNCs	0–0.4 mM	0.01 mM	[60]
Pd_150_-PCRP NPs	2–300 µM	1.65 μM	[61]
Pt/NiCo-LDH	50–500 mM	3.77 mM	[62]
Fe3O4/CND	0–20 µM	0.058 μM	[63]
**BC-AHE@Pd**	**1.16–1.67 mM**	**0.02114 mM**	**Present work**

## Data Availability

No new data were created or analyzed in this study. Data sharing is not applicable to this article.

## Supplementary Materials

The following supporting information can be downloaded at: https://www.mdpi.com/article/10.3390/molecules28186676/s1, Figure S1: GC-MS analysis of phytochemicals in *Artocarpus heterophyllus* seeds; Table S1: Qualitative analysis of phytochemicals in *Artocarpus heterophyllus* seeds; Figure S2: Images of the test results of qualitative analysis of phytochemicals; Table S2: Relative activity (%) of the BC-AHE@Pd nanocatalyst with respect to pH, catalyst loading, OPD and H_2_O_2_ concentrations; Equation (S1): Limit of Detection (LOD). References [64,65,66,67] are cited in Supplementary Materials.

## Sample Availability

Samples of the compounds are available from the authors.
