# Supplementary material for: Palladium Nanoparticles Grafted onto Phytochemical Functionalized Biochar: A Sustainable Nanozyme for Colorimetric Sensing of Glucose and Glutathione
Source: Molecules. 2023 Sep 18;28(18):6676. doi: 10.3390/molecules28186676 (PMC10537334; doi:10.3390/molecules28186676)
Supplement: Supplementary file 1 [file molecules-28-06676-s001.zip › molecules-2562177-supplementary.pdf]

Supporting Information

Palladium Nanoparticles Grafted onto Phytochemical Functionalized Biochar: A Sustainable Nanozyme for Colorimetric Sensing of Glucose and Glutathione

Aakhila Banu [1], Arnet Maria Antony [1], Balappa Somappa Sasidhar [2], Shivaputra A. Patil [3,*] and Siddappa A. Patil [1,*]

[1] Centre for Nano and Material Sciences, Jain (Deemed-to-be University), Jain Global Campus, Kanakapura, Bangalore 562112, Karnataka, India; aakhila.banu@jainuniversity.ac.in (A.B.); a.maria@jainuniversity.ac.in (A.M.A.)

[2] Chemical Sciences & Technology Division, National Institute for Interdisciplinary Science & Technology (CSIR-NIIST), Thiruvananthapuram 695019, Kerala, India; drsasidharbs@niist.res.in

[3] Pharmaceutical Sciences Department, College of Pharmacy, Rosalind Franklin University of Medicine and Science, 3333 Green Bay Road, North Chicago, IL 60064, USA

* Correspondence: shivaputra.patil@rosalindfranklin.edu (S.A.P.); p.siddappa@jainuniversity.ac.in (S.A.P.)

Table of contents

1. GC-MS analysis of phytochemicals in *Artocarpus heterophyllus* seeds --------------------- 3
2. Qualitative analysis of phytochemicals in *Artocarpus heterophyllus* seeds ---------------- 4
3. Images of the test results of qualitative analysis of phytochemicals ---------------------- 5
4. Relative activity (%) of the BC-AHE@Pd nanocatalyst with respect to pH, catalyst loading, OPD and H_2O_2 concentrations --- 6
5. Limit of Detection (LOD) -- 6
6. References -- 7

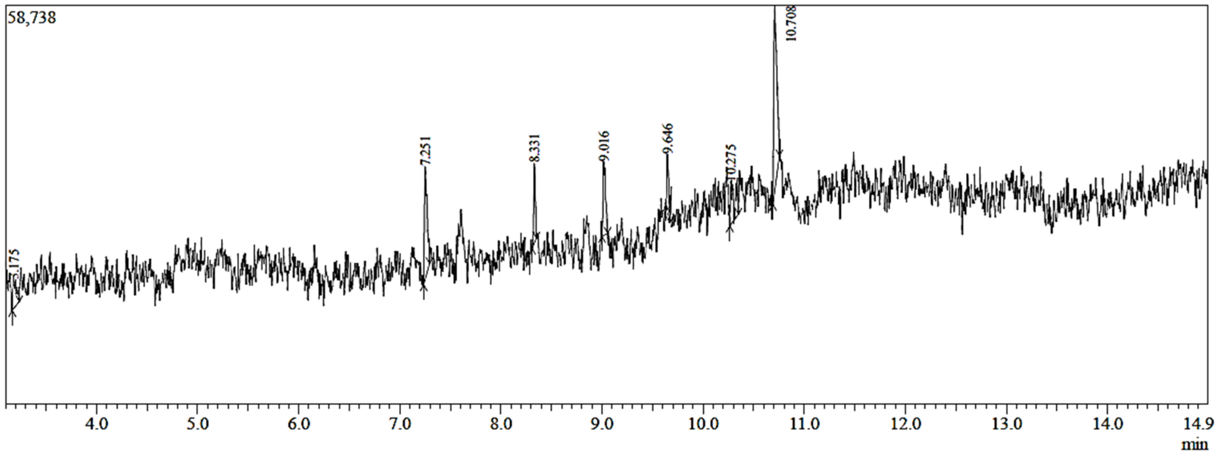

Compound name: 2-[3-(carbamoylmethyl)-2,4,6-trimethylphenyl]acetamide

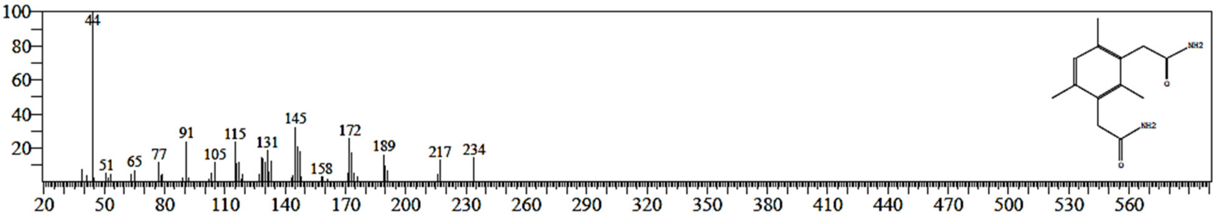

Compound name: 2,4-di-*tert*-butylphenol

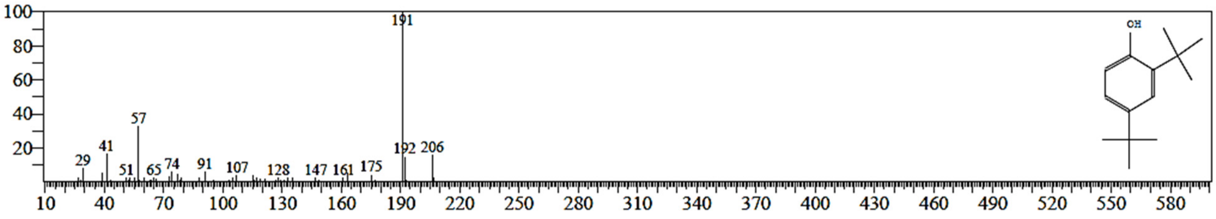

Compound name: 1-octadecanol

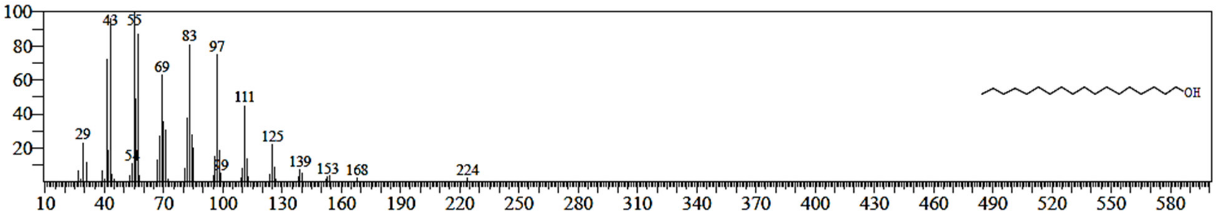

Compound name: 1-hexacosene

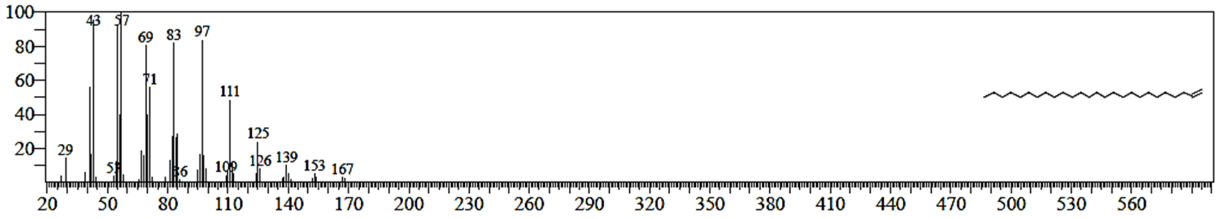

Compound name: ethylcyclooctadecane

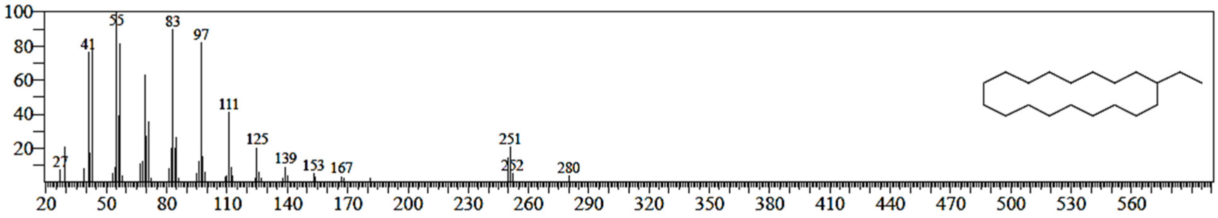

Compound name: bis(2-ethylhexyl) phthalate

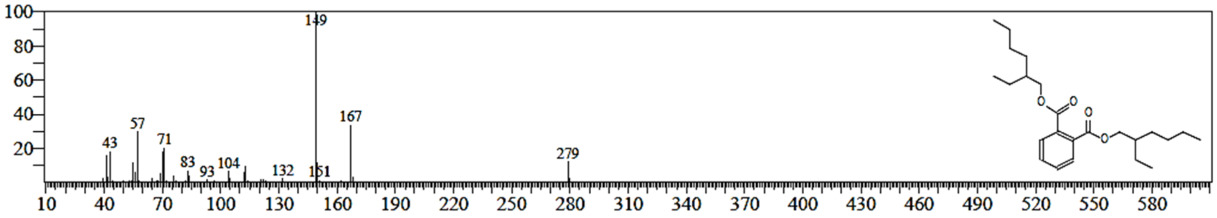

Figure S1. GC-MS analysis of phytochemicals in *Artocarpus heterophyllus* seeds.

Table S1. Qualitative analysis of phytochemicals in *Artocarpus heterophyllus* seeds [1, 2].

Sl. No.	Test	Observation	Inference
1.	**Test for saponins**		
	Plant extract (aq.) shaken with water in the test tube	Development of stable foam	Saponins are present
2.	**Test for sugars**		
	Plant extract + 2ml distilled water + Molish's reagent	Purple violet ring in interference	Sugars are present

3.	**Test for terpenoids**		
	Plant extract + few drops of conc. H_2SO_4	Formation of the pale yellow layer down	Terpenoids are present
4.	**Test for oxalate**		
	Plant extract + $KMNO_4$ + Conc. H_2SO_4	Decolorization	Oxalates are present
5.	**Test for glycosides and sterols**		
	Plant extract + 2 mL chloroform + Conc. H_2SO_4	Reddish brown interface formation	Glycosides and sterols are present

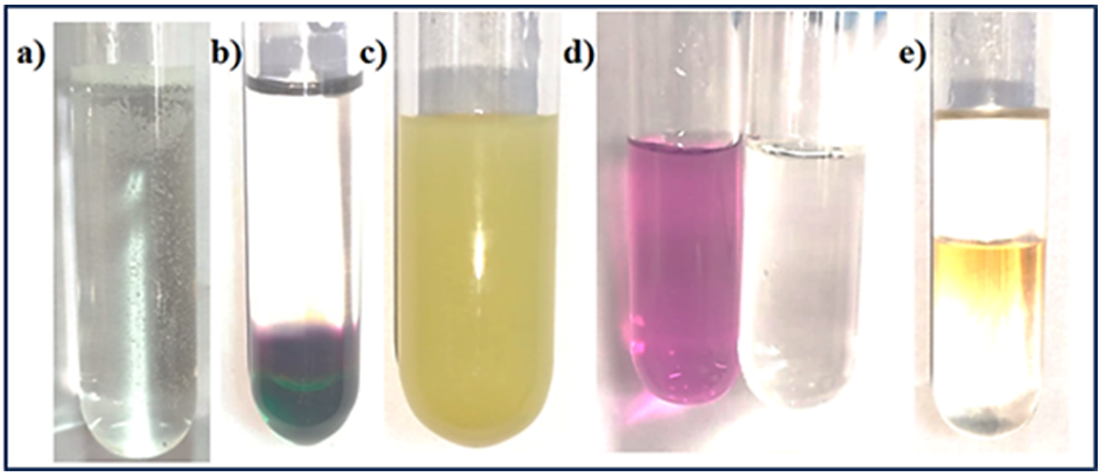

Figure S2. Images of the test results of qualitative analysis of phytochemicals.

Table S2. Relative activity (%) for the peroxidase-like activity of BC-AHE@Pd nanocatalyst with respect to pH, catalyst loading, OPD and H_2O_2 concentrations [3, 4].

**pH**	3	4	5	6	7	8	9
**Relative activity (%)**	53.52	100	36.43	20.61	6.68	6.38	6.13
**Catalyst loading (mol% Pd)**	0	0.10	0.16	0.21	0.26	0.31	
**Relative activity (%)**	2.49	67.09	75.03	82.22	91.80	100	
**OPD concentration (mM)**	0	0.1	0.8	0.6	0.4	0.24	
**Relative activity (%)**	0	21.72	23.47	32.11	38.62	100	
**H_2O_2 concentration (M)**	0	0.04	0.032	0.240	0.016	0.096	
**Relative activity (%)**	0	47.28	48.41	57.83	59.88	100	

Equation S1. Limit of Detection (LOD).

LOD= 3.3 S

LOD for H_2O_2

Standard deviation (0.03652 slope of the calibration curve (S) = 1.08105

$$\text{LOD}= \frac{3.3 \times 0.03652}{1.08105} = 0.1115 \text{ M}$$

LOD for OPD

Standard deviation (slope of the calibration curve (S) = 3.35072

$$\text{LOD}= \frac{3.3 \times 0.08162}{3.35072} = 0.0804 \text{ mM}$$

References

1. Ojwang, R.; Muge, E.; Mbatia, B.; Mwanza, B.; Ogoyi, D., Comparative Analysis of Phytochemical Composition and Antioxidant Activities of Methanolic Extracts of Leaves, Roots and Bark of Jackfruit (Artocapus heterophyllus) from Selected Regions in Kenya and Uganda. *J. Adv. Biol. Biotechnol.* **2017,** 16.
2. Sreeja Devi, P. S.; Kumar, N. S.; Sabu, K. K., Phytochemical profiling and antioxidant activities of different parts of Artocarpus heterophyllus Lam. (Moraceae): A review on current status of knowledge. *FJPS* **2021,** 7, 30.
3. Jeon, H. J.; Kim, H. S.; Chung, E.; Lee, D. Y., Nanozyme-based colorimetric biosensor with a systemic quantification algorithm for noninvasive glucose monitoring. *Theranostics* **2022,** 12, 6308-6338.
4. Xian, Z.; Zhang, L.; Yu, Y.; Lin, B.; Wang, Y.; Guo, M.; Cao, Y., Nanozyme based on CoFe2O4 modified with MoS2 for colorimetric determination of cysteine and glutathione. *Mikrochim. Acta* **2021,** 188, 65.
